# Commentary: Are Children Like Werewolves? Full Moon and Its Association with Sleep and Activity Behaviors in an International Sample of Children

**DOI:** 10.3389/fped.2016.00061

**Published:** 2016-06-09

**Authors:** Chris Fradkin, Christophe Huynh

**Affiliations:** ^1^University of California Merced, Merced, CA, USA; ^2^Centre de réadaptation en dépendance de Montréal – Institut universitaire, Montreal, QC, Canada

**Keywords:** moon, lunar cycle, sleep, physical activity, children, myth-busting

A recent article by Chaput and colleagues (1) provides evidence that if a relationship exists between lunar cycle and sleep duration and physical activity in children, its association is rather weak and less-than-meaningful. Using an international sample of 9- to 11-year olds (*n* = 5812), monitored by accelerometers, Chaput and his team found no relationship between lunar cycle and physical activity, and a minimal effect between lunar cycle and sleep duration (~5 min/night less sleep under full moon vs. new moon). While this relationship has been studied in the past, this is the first time it has been studied among a diverse pediatric sample of this size.

The authors’ findings are important because they provide further evidence against the notion of a so-called *lunar effect*, a phenomenon that purports increased violence, crime, and birth-rate, occurring in the full moon phase. While this concept may seem far-fetched to some, to many people it is not. In fact, according to a survey of 325 people (2), 140 (43%) thought that “lunar phenomena could alter personal behavior” [(3), p. 149]. Interestingly, this belief was most popular among the health professionals, including social workers, clinical psychologists, and nurses’ aides, surveyed in the sample. A separate survey was consistent with these findings. In an examination of lunar cycle on emergency medicine personnel (*n* = 50), Danzl (4) found that 80% of the emergency department nurses and 64% of the physicians believed that lunar cycle impacts patients. Among the nurses, 92% found higher stress on lunar shifts and expressed desire for financial compensation. Though these anecdotal findings have been empirically debunked (5–11), the portent of the full moon lingers on.

To date, the literature on lunar cycle–sleep relationship has been inconsistent in its findings. Among a sample of adults (*n* = 205), Della Monica and colleagues (12) found a lunar class × sex interaction, with women with reduced sleep, and men with increased REM sleep, in relation to the full moon phase. A study by Turányi and colleagues (13) found reduced deep sleep and sleep efficiency, occurring at the full moon phase. In contrast, several studies (14, 15) have shown no sleep effect at all, occurring at the full moon phase. And in one of the few studies using children (*n* = 795), Sjödin and colleagues (16) found longer sleep length and reduced activity, both occurring at the full moon phase. With respect to these works, the study by Chaput and colleagues (1) is the largest of its kind, in terms of sample size and geographic scope.

Another strength of the Chaput and colleagues study (1) is its interpretation of statistical results. Although the association between lunar cycle and sleep duration was *statistically significant* at *p* < 0.01, the authors actually point out that such a result, i.e., a 5-min reduction at full moon compared with new moon, is *not clinically relevant*. They explain correctly that a large sample size tends to increase the likelihood of finding a statistically significant result (17, 18). Their discussion demonstrates an intellectual rigor that needs to be highlighted, because too often it is tempting to interpret a statistically significant result as a meaningful and relevant discovery. Rather, Chaput and his team downplay their findings – findings that could have been interpreted as a robust relationship between lunar cycle and sleep duration.

In their closing remarks, Chaput and colleagues (1) stress their need to delve much deeper, in terms of future research. In particular, they mention testing the relationship between the full moon on subgroups of vulnerable children, including “those with mental disorders and physical ailments” [(1), p. 5]. While this is an expected course to take, the authors should remember that sleep problems are associated with many physical ailments, which in turn can be cormorbid with mental disorders (19). What is needed, therefore, is a better understanding of the impact of mental and physical comorbidities on sleep parameters as potential third and confounding variables. We recommend this step *prior* to the proposed testing of the lunar cycle–sleep relationship among samples of vulnerable children. But regardless of their tack, movement forward with this work has the potential to unearth interesting findings, in particular, if the authors have a sample of the size and the diversity as the one they used in this study.

Another aspect of this study worth mentioning again is its geographical diversity in sampling. It is unusual to encounter a study that has such diversity in sampling, not just racially, ethnically, and economically but also geographically. The authors’ finding of no differences across the study sites (i.e., geographically) suggests the findings are consistent, independent of location, for children in this age group (9–11 years) around the world.

In conclusion, Chaput and colleagues (1) have moved us one step closer, in our understanding of lunar cycle, sleep, and activity. Other methods of assessment, such as forced desynchrony protocols (20), will allow exploring other questions on the impact of lunar cycle on behavior. These methods may contribute to debunking long-held myths, or possibly confirming lay beliefs.

## Author Contributions

Both authors contributed to the content, writing, and review of this commentary.

## Conflict of Interest Statement

The authors declare that the research was conducted in the absence of any commercial or financial relationships that could be construed as a potential conflict of interest.
